# The Value of Aortic Volume and Intraluminal Thrombus Quantification for Predicting Aortic Events after Endovascular Thoracic Aneurysm Repair

**DOI:** 10.3390/jcm13102981

**Published:** 2024-05-18

**Authors:** Mariangela De Masi, Carine Guivier-Curien, Sébastien Cortaredona, Virgile Omnes, Laurence Bal, Baptiste Muselier, Axel Bartoli, Marine Gaudry, Philippe Piquet, Valérie Deplano

**Affiliations:** 1 Timone Aortic Center, Department of Vascular Surgery, APHM, Timone Hospital, 13005 Marseille, France; virgile.omnes@ap-hm.fr (V.O.); laurence.bal@ap-hm.fr (L.B.); marine.gaudry@ap-hm.fr (M.G.); philippe.piquet@ap-hm.fr (P.P.); 2 CNRS, École Centrale Marseille, IRPHE UMR 7342, Aix-Marseille University, 13384 Marseille, France; carine.guivier@univ-amu.fr (C.G.-C.); valerie.deplano@univ-amu.fr (V.D.); 3 IRD, AP-HM, SSA, VITROME, Aix-Marseille University, 13009 Marseille, France; sebastien.cortaredona@ird.fr; 4 IHU-Méditerranée Infection, 13385 Marseille, France; 5 Department of Radiology, APHM, Timone Hospital, 13005 Marseille, France; baptiste.muselier@ap-hm.fr (B.M.); axel.bartoli@ap-hm.fr (A.B.)

**Keywords:** thoracic aortic aneurysm, volume analysis, predictive factors, long term results

## Abstract

**Objectives:** To assess the ability of the aortic aneurysm volume (AAV), aneurysmal lumen volume (ALV), and aneurysmal thrombus volume (ATV) to predict the need for aortic reintervention when using the maximal aortic diameter as a reference. **Methods:** This monocentric retrospective study included 31 consecutive patients who underwent successful thoracic endovascular aortic repair (TEVAR) to treat an atheromatous thoracic aortic aneurysm. All patients underwent clinical and computed tomography angiography (CTA) for 3 years after TEVAR. The patients were categorized into group 0 if no aortic reintervention was required during the follow-up period and categorized into group 1 if they experienced a type I or III endoleak or aneurysm diameter increase requiring intervention. The maximum aneurysm sac diameter and the AAV, ALV, and ATV were calculated using CTA images obtained preoperatively (T0) and at 6–12 months (T1), 24 months (T2), and 36 months (T3) postoperatively, and their changes over time were analyzed. Correlations between diameter and changes in AAV, ALV, and ATV were assessed, and the association between diameter and volume changes and reintervetion was examined. The cutoff values for predicting the need for reintervention was determined using a receiver operating characteristic (ROC) curve. The accuracy of volume change versus diameter change for predicting the need for reintervention was analyzed. **Results:** There were no significant differences in terms of the mean aneurysm diameter or AAV, ALV or ATV between the groups at preoperative CTA or after one year of follow-up imaging. The mean ATV was higher in group 1 than in group 0 at 2 years (187.6 ± 86.3 mL vs. 114.7 ± 64.7 mL; *p* = 0.057) and after 3 years (195.0 ± 86.7 mL vs. 82.1 ± 39.9 mL; *p* = 0.013). The maximal diameter was greater in group 1 than in group 0 at 3 years (67.3 ± 9.5 mm vs. 55.3 ± 12.6 mm; *p* = 0.044). The rate of AAV change between T0 and T1 was significantly higher in group 1 (7 ± 4.5%) than in group 0 (−6 ± 6.8%; *p* < 0.001). The rate of ATV change between T1-T3 was significantly higher in group 1 than in group 0 (34 ± 40.9% vs. −13 ± 14.4% (*p* = 0.041)); similar results were observed for the rate of ATV change between T2 and T3 (27 ± 50.1% for group 1 vs. −8 ± 49.5% in group 0 (*p* < 0.001)). According to our multivariate analysis, the annual growth rate for AAV between T0 and T1 was the only independent factor that was significantly associated with aortic reintervention (area under the curve (AUC) = 0.84, OR = 1.57, *p* = 0.025; optimal cutoff +0.4%). An increase in the annual growth rate of the ATV between T0 and T3 was independently associated with the need for aortic reintervention (area under the curve (AUC) = 0.90, OR = 1.11, *p* = 0.0347; optimal cutoff +10.1%). **Conclusions:** Aortic volume analysis can predict the need for aortic reintervention more accurately and earlier than maximal aortic diameter.

## 1. Introduction

Thoracic endovascular aortic repair (TEVAR) has been proposed to be a less invasive technique for the treatment of thoracic aortic aneurysms. Early clinical results with thoracic aortic stent grafts have shown significantly improved early quality of life versus open surgery and have generally shown a trend toward better perioperative survival and freedom from major complications [1,2]. Procedural technical success is achieved when the endograft is deployed accurately and the aneurysm is excluded from the circulation due to the absence of type I or III endoleaks. Evaluation of clinical success requires follow-up examinations showing complete thrombosis and shrinkage of the aneurysm sac. The main drawback of this technique is that follow-up is needed after TEVAR to evaluate whether long-term complications, such as migration, aneurysm expansion with or without endoleaks, device failure (fracture, migration, or component separation), stenosis, or occlusion, occur [3,4]. The most commonly reported follow-up protocols after TEVAR for aneurysms are clinical examination and computed tomography angiography (CTA) at 1 month, 6 months, and yearly thereafter [5,6]. Meena et al. reported aorta-related complications in 35% of patients (72/203), with sac expansion accounting for 77% of them [7]. Ziza et al. showed in a series of 82 patients treated for thoracic aortic aneurysm (TAA) that 11% underwent reintervention for direct endoleaks (7%) [3]. A recent review of the outcomes revealed that 77/144 (53.5%) patients treated for intact aortic aneurysms required reintervention within 90 days of TEVAR [8]. Baldaia et al. confirmed that TEVAR is a first line treatment for thoracic aortic disease but required the identification of possible morphological factors of worse outcomes [9]. The risk of endograft failure after TEVAR was not similar in all patients. Some patients developed endoleaks, which lead to aortic-related death due to rupture and necessitated reintervention, including late open conversion [10]. Previous reports have described the morphological changes of aneurysms based on only the maximum diameter measured from the axial and/or longitudinal view. This measurement reflects only linear changes in a single cross-sectional area. The maximum aortic diameter is an accepted surrogate marker of the risk of aortic rupture owing to increased wall tension with increased radial growth [11]. On the other hand, the use of aortic diameter as a surrogate marker has several limitations, and aortic volume is better than aortic diameter because it reflects the three-dimensional (3D) morphological changes in the aneurysm [12,13]. Aortic volume measurement is now used widely in the follow-up of EVAR to evaluate the behavior of the aneurysm; many authors consider this parameter much more reliable than the simple maximum diameter measurement [14,15].

Although aortic thrombus is routinely assessed as part of preoperative planning, a standardized measurement for evaluating the volumetric modification of thrombus volume has not been reported. Studies of volumetric analysis following TEVAR to treat TAA are scarce and have mostly been conducted following thoracic aortic dissection [16,17]. Aortic and thrombus volume quantification and evolution over time may be more accurate and serve as early surrogate markers for predicting aortic remodeling and determining the best time for reintervention.

In the present study, semiautomated software was used to quantify aortic aneurysmal volume (AAV), aneurysmal lumen volume (ALV), and aneurysmal thrombus volume (ATV) based on elementary volume addition [16,18,19]. The aim of the present study was to compare the ability of AAV, ALV, and ATV to predict the need for thoracic aneurysm reintervention after successful TEVAR, with the aortic maximal diameter (D) used as a reference.

## 2. Materials and Methods

### 2.1. Design and Population

This was a single-center, retrospective study on patients who underwent TEVAR from 2006 to 2022 for chronic non-dissecting thoracic aortic aneurysmal lesions at the Vascular Surgery Department of Timone Hospital. This study was approved by the local institutional review board, and the need for informed consent for the study was waived (PADS23-17). Patient demographics, comorbidities, intraoperative data as well as pre- and postoperative computed tomographic angiogram (CTA) findings (maximum aortic diameter and landing zone) and complications post-TEVAR were analyzed.

In our study, the inclusion criteria were as follows: chronic degenerative aneurysm that required treatment with thoracic stent grafts; only patients with proximal landing zone of the stent graft in Ishimaru zone 2, 3, or 4 of the thoracic aorta; preoperative contrast-enhanced computed tomography angiography (CTA) within 6 months of the index surgery (T0) and three postoperative follow-up CTA: the first approximately 6–12 months after surgery (T1) and the second and third within 24 months (T2) and 36 months (T3) of TEVAR, as per the institutional protocol. The exclusion criteria were as follows: patients who did not have follow-up in each of the three years following TEVAR, signs of aortitis or periaortitis, infectious aneurysms, aortic dissection and contraindications to CTA. The data of any patient who required aortic reintervention were not included in the study after reintervention.

### 2.2. Data Management

All clinical data were collected before the intervention and at each follow-up visit. CTA images were acquired before the intervention and annually thereafter for three years. Postoperative CTA was first used to determine whether TEVAR was successful and whether the thoracic aortic lesion was completely excluded. All patients included in this study were divided retrospectively into two groups, with and without aortic reinterventions: group 1 (*n* = 10), which included all patients who required aortic reintervention during the 3-year follow-up period, and group 0 (*n* = 21), which included patients who did not require aortic reintervention. Aortic reintervention was performed to resolve type I or III endoleaks following proximal or distal stent-graft implantation or type II endoleaks following embolization of the side branch vessels. CTA images of poor quality or obtained after reintervention were excluded from the database. Three types of commercially available stent grafts were used: TAG (W. L. Gore and Associates, Inc., Flagstaff, AZ, USA), Zenith TX2 (Cook Medical, Inc., Bloomington, IN, USA), and Valiant (Medtronic, Inc., Santa Rosa, CA, USA). The choice of device was based on the expertise of the vascular surgeon. TEVAR was performed in an operating room under local or general anesthesia following best medical practices at the time of the surgery.

### 2.3. Image Analysis and Measurement Volume

**Figure 1:**
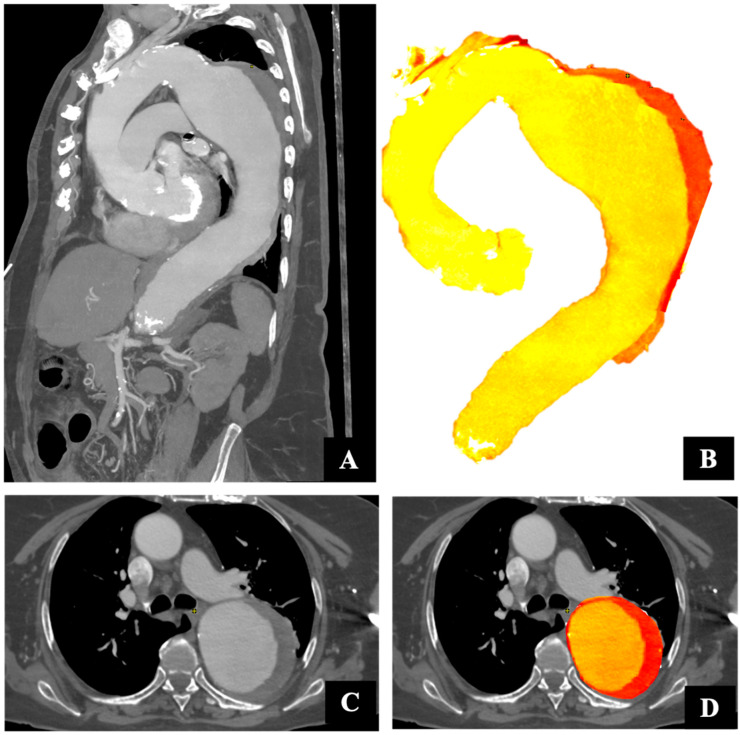
Thoracic aortic volume. Preoperative sagittal (**A**,**B**) and axial (**C**,**D**) computed tomography images. The yellow area indicates the aortic lumen, and the red area indicates the thrombus and wall.

**Figure 2:**
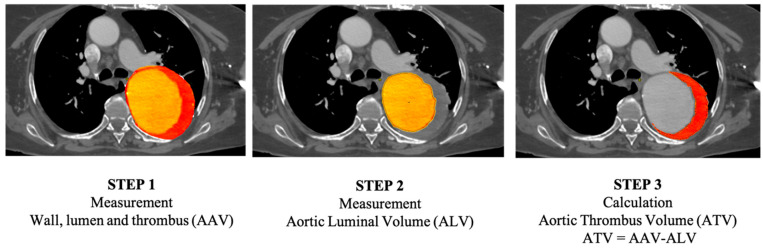
Volume measurement. Step 1. Measurement of aortic volume: wall, intraluminal thrombus (red area), and aortic lumen (yellow area). Step 2: Measurement of the aortic luminal volume (yellow area). Step 3: Calculation of the residual volume corresponding to intraluminal thrombus volume.

All CTA measurements were obtained using semiautomatic reconstruction software (EndoSize ® 3.1.40; Therenva, Rennes, France). The maximum aneurysm diameter was measured on images obtained perpendicular to the centerline (CL) of the aorta, measuring the outer wall to the outer wall. All measurements were recorded in millimeters (to the nearest 0.1 mm). We analyzed the changes in aortic diameter and volume between the preoperative (T0) images and the 1- (T1), 2- (T2), and 3-year (T3) postoperative CTA images. Volumetric quantification of the aorta was achieved with a custom application, named thoracic aorta volume (TAV-IRPHE), as previously reported and validated [16]. Briefly, to quantify several aortic volumes, we used the enhanced part of the aorta to define the aortic lumen. Aortic thrombus was defined as all of the non-enhanced tissues included within the aortic wall. The superior limit of the aortic aneurysm was defined as the distal part of the proximal neck, and the distal limit of the aneurysm was defined as the proximal part of the distal neck. The maximal aortic diameter (D) and the differences in AAV, ALV, and ATV were quantified and analyzed. We also studied the total thoracic aorta volume in patients with and without thrombus (Figure 1). The total aortic volume included the wall of the aorta (intima, media, and adventitia), aortic lumen, and ATV. The ATV was calculated as a percentage of the residual aortic volume of the aneurysm ATV = [(ATV/AAV) × 100] (Figure 2). The mean annual growth rate (%) (i.e., geometric mean) for each time point and each variable was calculated.

Patient comorbidities and aneurysm-related outcomes were reported according to specified guidelines [5]. Aneurysm sac expansion was assessed between the pre- and each postoperative CTA image. Sac growth was defined as an increase in aneurysm sac diameter > 5 mm/year. An endoleak was defined as any radiological evidence of blood circulation in the aneurysmal sac [20]. TAA-related complications were defined as a composite of the following: type I or III endoleak, type II endoleak, migration, or infection. The primary endpoint was the need for aortic reintervention due to an increase in diameter > 5 mm/year or a type I or III endoleak.

### 2.4. Statistical Analysis

Continuous variables are presented as the mean and standard deviation (SD) or as the median and interquartile range (IQR). The mean annual growth rate (%) (i.e., geometric mean) for each time point and each variable was calculated. The formula used is:

$$t=\left({\left(\frac{Q^{\prime }}{Q}\right)}^{\frac{1}{n}}-1\right)$$

*Q′*: value in the later period.

*Q*: value in the early period.

*n* = the number of periods between the earlier period and the later period.

The chi-squared test or Fisher’s exact test was used for comparisons of categorical variables between groups, as appropriate. The Mann–Whitney–Wilcoxon test was used to compare differences in the distribution of the values. Spearman’s rank correlation analysis (Rs = ρ) was conducted to estimate correlations between AAV, ALV, and ATV and the maximum diameter. The values of coefficient ρ are positive or negative: coefficients between 0.40 and 0.59 indicate a moderate correlation, coefficients between 0.70 and 0.89 indicate a strong correlation, and coefficients between 0.90 and 1.00 indicate a very strong correlation. A *p*-value < 0.05 was considered to indicate statistical significance. Receiver operating characteristic (ROC) curve analyses and the Youden index were used to evaluate whether the parameters were predictive of the need for TAA reintervention. Multivariate logistic regressions were also performed with TAA reintervention as the outcome. Three separate models were used: one with variables measured between T0 and T1, another with variables measured between T0 and T2, and a final one with variables measured between T0 and T3. All statistically significant parameters identified in the univariate analysis (baseline values and growth rates for D, AAV, ALV, ATV, and total thoracic aorta, both with and without thrombus) were chosen as candidate variables for the multivariable analysis, employing a stepwise selection method. As parameter estimates of logistic regressions can be biased if the number of events is small, as in our study, we used Firth’s correction to reduce possible bias in estimation [21,22,23]. For all analyses, two-sided *p*-values ≤ 0.05 were considered to indicate statistical significance. Statistical analyses were performed with SAS (version 9.4, SAS Institute, Cary, NC, USA).

## 3. Results

### 3.1. Baseline Characteristics

The study population included 31 patients who underwent TEVAR for aortic degenerative aneurysm. A total of 128 CTA images were reviewed, and 92 were used for analysis. The patients had a mean (SD) age of 80.2 years (12.4) and a median [Q1, Q3] age of 81.5 years [79.5, 86.5]. The group without intervention was younger (79.1 ± 14.5 vs. 82.3 ± 6.9) and had a higher proportion of cardiac artery disease and hypertension than the group with post-TEVAR reintervention. All baseline demographic data are shown in Table 1.

The perioperative characteristics and common indications for reintervention are summarized in Table 2. Technical success of the first TEVAR intervention was achieved in 100% of cases in both group according to the European Society of Vascular Surgery criteria [5,24]. A debranching procedure was performed in 12 cases (38%), with 100% transpositions of the left subclavian artery and 46% debranching of the left carotid artery.

### 3.2. Reinterventions

Twenty-one patients in group 0 did not require reintervention during the follow-up period. Ten patients experienced aortic complications (Type I endoleak I, *n* = 5; II, type II endoleak *n* = 2; type III endoleak, *n* = 3) and required reintervention during the first three years of follow-up. Endovascular treatment options for patients with type I and III endoleaks include aortic extension. Secondary procedures were performed for one patient 12 months after, for 4 patients 24 months after, and for 5 patients 36 months after elective TEVAR.

### 3.3. Thoracic Aortic Aneurysm Sac Comparison

There were no significant differences in terms of the mean aneurysm diameter or AAV, ALV, or ATV between the groups on preoperative CTA or after one year of follow-up. On the other hand, the mean ATV was higher in group 1 than in group 0 at 2 years (187.6 ± 86.3 vs. 114.7 ± 64.7 mL; *p* = 0.057) and at 3 years (195 ± 86.7 vs. 82.1 ± 39.9 mL; *p* = 0.013) of follow-up. After 3 years of follow-up, D was greater in group 1 (67.3 ± 9.5 mL) than in group 0 (55.3 ± 12.6 mL; *p* = 0.044) (Table 3).

### 3.4. Diameter and Volume Mean Annual Growth Rates

The changes in diameter and aortic volume are shown in Table 4.

The mean annual growth rate of AAV was significantly greater in group 1 (6.63 ± 4.49%) than in group 0 (−6.31 ± 6.83%; *p* < 0.001) between T0 and T1. This difference remained significant for later time points (T2 and T3). This result showed that AAV is the earliest parameter showing a significant difference between groups. There was no significant difference in the annual growth rate of ATV between T0 and T1 (+32.8 ± 99.86% vs. +42.8 ± 101.18%), but it was significantly higher in group 1 than in group 0 (T3-T1), with an annual growth rate of +34 ± 40.92% in group 1 versus −13 ± 14.42% in group 0 (*p* = 0.041), and between T3 and T2, with an annual growth rate of +27 ± 50.07% for group 1 versus a rate of −8 ± 49.54% in group 0 (*p* < 0.001). The maximum diameter annual growth rate between T2 and T1 was significantly greater for group 1 than for group 0 (6 ± 9.72 versus −3 ± 6.04%, respectively (*p* = 0.013)).

Spearman’s rank correlation analysis showed that there was no correlation between the maximal diameter of the aneurysm and ATV at any time point. On the other hand, there was a significant correlation between D and AAV but only at T3 (ρ = 0.70; *p* = 0.004). Spearman’s rank correlation showed a moderate correlation between D and AAV at T0 (ρ = 0.43; *p* = 0.001), T1 (ρ = 0.45; *p* = 0.001), and T2 (ρ = 0. 52; *p* = 0.001). We found a moderate correlation between the maximal D and ALV at the preoperative time (ρ = 0.45; *p* = 0.001) and at T2 (ρ = 0. 52; *p* = 0.001).

**Figure 3:**
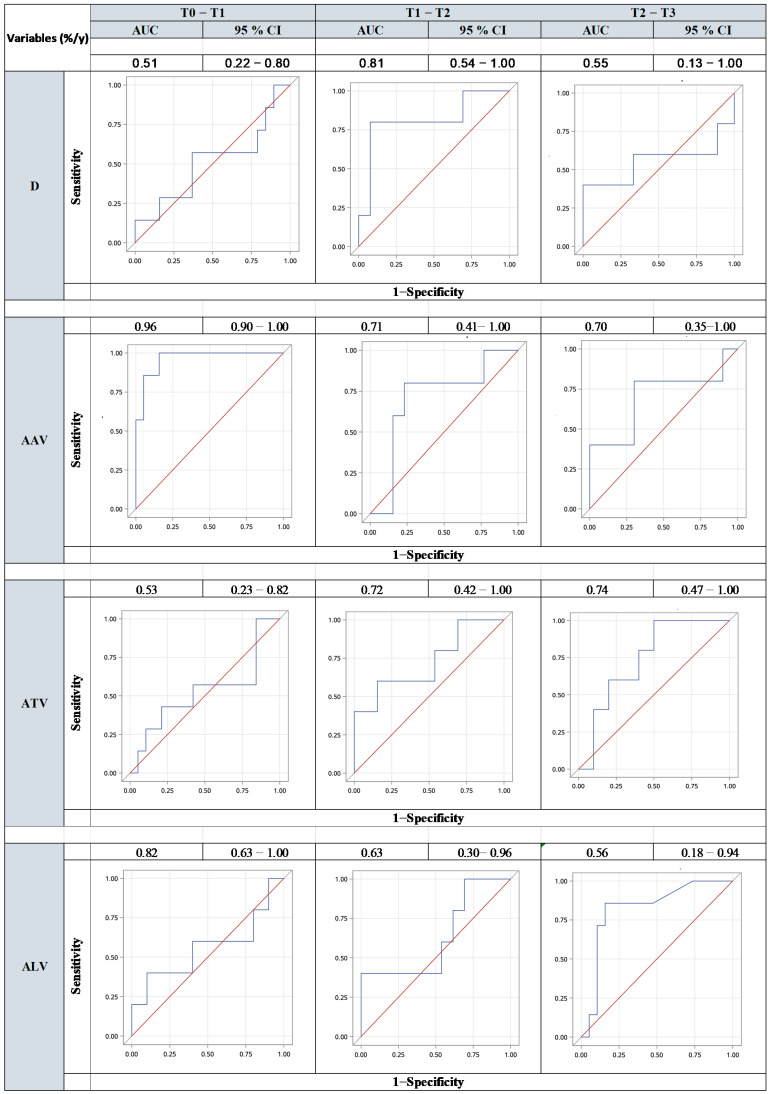
Receiver operating characteristic (ROC) curve analysis between two time points, diameter (D), aortic aneurysmal volume (AAV), aneurysmal thrombus volume (ATV), and aneurysmal lumen volume (ALV) to predict the need for reinterventions.

To further compare the ability of D and volume to predict the need for reinterventions, ROC curve analyses were performed between two time points (i.e., the mean annual growth rate between two time points) for the following parameters: D, AAV, ATV, and ALV (Figure 3). AAV predicts the risk of reinterventions after *TEVAR* at the earliest time point, as indicated by the highest area under the curve value (T0-T1 (area under the curve (AUC) 0.96, 95% CI 0.90–1.00)) (Figure 3. Between T1 and T2 and between T2 and T3, the results were similar for AAV, with AUCs of 0.71 (95% CI 0.41–1.00) and 0.70 (95% CI 0.35–1.00), respectively. The change in ALV between T0 and T1 had an AUC of 0.82 (95% CI 0.63–1.00). The change in ATV between T1 and T2 predicted reintervention with an AUC of 0.72 (95% CI 0.42–1.00; similar results were obtained for the change in ATV between T2 and T3 (AUC 0.74, 95% CI 0.47–1.00). On the other hand, D significantly predicted reintervention after *TEVAR* between T1 and T2 (AUC 0.81, 95%, CI 0.54–1.00).

Ten patients required TAA reintervention for endoleaks. After testing simultaneously for all geometrical parameters studied (baseline values and growth rates for D, AAV, ALV ATV, and the total thoracic aorta with and without thrombus) in a stepwise multivariate model, the annual growth rate for AAV was the only independent factor significantly associated with the need for aortic reintervention between T0 and T1 (AUC = 0.84, OR = 1.57, *p* = 0.025, optimal cutoff +0.4%) and between T0 and T2 (AUC = 0.86, OR = 1.14, *p* = 0.0498, optimal cutoff +7.3%). Preoperative thrombus burden was not associated with a higher risk of reintervention, but an increase in the annual growth rate of ATV between T0 and T3 was significantly associated with the need for aortic reintervention (AUC = 0.90, OR = 1.11, *p* = 0.0347; optimal cutoff +10.1%).

## 4. Discussion

The main findings of the study are as follows: (1) in patients who benefit from TEVAR for the treatment of degenerative TAAs, the early change in AAV is a more accurate predictor of aortic events than yearly changes in diameter; (2) ALV seems to have a weaker predictive value than AAV; (3) an annual growth rate of AAV higher than 7% predicts aortic reintervention with an accuracy of 96.9%, a sensitivity of 100% (68.4–100%), and a specificity of 84%. (57.1–100%); and (4) preoperative thrombus burden was not associated with a higher risk of endoleaks, but a more than 10% increase in ATV during the follow-up period predicts the need for aortic reintervention with an accuracy of 90%, a sensitivity of 83% (42.8–100%), and a specificity of 90% (77.1–100%).

The current guidelines offer an example of a follow-up algorithm after TEVAR for aneurysms, with the first postoperative CTA scan recommended at 1 month, after 6 months, and yearly thereafter [5]. Atherosclerotic aneurysms are known to be the most difficult to treat because they do not have a sharp demarcation against healthy aortae. Over time, the disease progresses to the neck, resulting in endoleaks [26]. During the follow-up period, aortic diameter is considered to be the gold standard parameter used in clinical decision-making, while volume measurement is studied but not used due to the time required to perform such measurements in clinical practice. In the present study, the quantification and evolution of aortic aneurysm and thrombus volume over time may be markers in addition to diameter for predicting aortic remodeling and determining the best time to propose reintervention.

Our study only included patients with complete follow-up data. The patients were divided into group 0 (21 patients; 67.74%) and group 1 (10 patients; 32.25%). Aneurysm expansion occurred in a significant number of patients following TEVAR for TAA, showing that these patients remain at risk of aortic rupture after TEVAR. Approximately 35% of patients experienced sac expansion within 3 years of stent graft implantation. We noted that the diameter increased during the two or even three years of follow-up, whereas the volume seemed more sensitive and changed from the first CTA scan. The rate of change in the maximum diameter did not significantly differ between groups between T0 and T1 because the diameter decreased by 4% in all patients in each group. In contrast, the rate of AAV growth between T0 and T1 was significantly greater in group 1 than in group 0, with a median rate of change of +7% versus −3%, respectively (*p* < 0.001). Therefore, our patients presented an increase in aortic volume as soon as the first follow-up scan, despite a stable maximum aortic diameter. In fact, the maximal diameter only represents a small section of the aneurysm, while the majority of changes in size involve the aneurysm sac as a whole. Therefore, volume is considered to be the parameter that most strongly reflects morphological aneurysm changes. Aneurysm volumetric analysis for the evaluation of aortic outcomes is a very precise but time-consuming measurement that is currently not applicable in clinical practice. Artificial intelligence techniques open new horizons for such measurements in the clinical field. In particular, in the presence of type II endoleaks, it is more difficult to determine the real morphologic changes during the follow-up period, and volume measurements could help clinicians ascertain any differences. In most circumstances, type II endoleaks are typically classified as benign and therefore managed conservatively because there are minimal or show no changes in diameter. Most type II endoleaks spontaneously thrombose but are not always benign. In our series, type II endoleaks were treated as persistent endoleaks with aneurysm sac expansion, with a risk of rupture. Bischoff et al. [27] also reported that 9 of 30 patients with persistent type II endoleaks after TEVAR required reintervention. The procedures were performed in nine patients, with seven undergoing endovascular treatment and two requiring late conversions. Bischoff et al. [27] observed type II endoleak-mediated fatal aneurysm ruptures, both of which illustrated potential challenges associated with type II endoleaks. Our detailed analysis of patients who underwent reoperation revealed that a stable aneurysm sac or a slightly enlarged aneurysm diameter can be associated with a poor prognosis.

Another aneurysm characteristic related to endoleaks in our study was preoperative and postoperative thrombus volume. To the best of the authors’ knowledge, this is the first study to focus on the impact of the total volume of thoracic aneurysms and the free space occupied by thrombi during the three years of follow-up after TEVAR. A statistically significant difference in the ATV between group 0 and group 1 was observed at T3 (*p* < 0.002). No significant correlation between the ATV and the diameter of the thoracic aneurysm aortic sac was found in the present study. The present study showed that the ATV represented 32% of the total volume in group 0 and 42% of the total volume in group 1 preoperatively. An increase in the thrombus burden during the follow-up period was associated with a higher risk of endoleaks. Aortic thrombus has been correlated with the biological expression of both D-dimer and neutrophil elastase-derived cross-linked fibrin degradation products [28,29]. The present study demonstrated that an increase in the annual growth rate of aneurysm thrombus volume was associated with an increased risk of reintervention. These data support the notion that the thrombus is a biologically active tissue, and any change in sac size is directly related to changes in the thrombus of the aneurysmal sac [30,31]. In addition, Polzer [28] and Riveros [32] noted that under increased stress in the thrombus, small fractures may develop, providing additional compartments for proteolytic activity. However, to our knowledge, there is no literature on how thrombus volume changes after TEVAR and why, according to our results, a greater ATV impedes sac regression. Concerning EVAR, conflicting results [15,33,34] have been reported regarding different aspects of thrombus burden (size and volume) and localization. The biomechanical characteristics of the thrombus may therefore be important in relation to sac changes after TEVAR.

This study has several limitations. It is retrospective and included only a small number of patients with a mid-term follow-up duration. Future studies with a larger sample of patients with or without reintervention and longer follow-up are needed to validate these results.

## 5. Conclusions

In patients who benefit from TEVAR for the treatment of degenerative thoracic aortic aneurysms, the annual growth rate of aortic aneurysm volume is more accurate to predict aortic events than the annual growth rate of aortic aneurysm diameter. An annual growth rate of aortic aneurysm volume higher than 7% predicts aortic reintervention with an accuracy of 96.9%, sensitivity of 100% (68.4–100%), and specificity of 84% (57.1–100%). 

## Figures and Tables

**Table 1:** Clinical characteristics.

Variables	Group 0 No Thoracic Aortic Aneurysm Reintervention	Group 1 Thoracic Aortic Aneurysm Reintervention	All	*p*-Value
	n = 21 (67.7%)	n = 10 (32.3%)	n = 31 (100.0%)	
	Mean ± SD	Mean ± SD	Mean ± SD	
Age, years	79.1 ± 14.5	82.3 ± 6.9	80.2 ± 12.4	0.862
Height, cm	169.5 ± 6.9	171.0 ± 8.2	170.0 ± 7.3	0.701
Weight, kg	71.9 ± 17.0	74.7 ± 15.9	72.9 ± 16.4	0.523
ASA n	2.9 ± 0.5	3.1 ± 0.5	3.0 ± 0.5	0.531
	n (%)	n (%)	n (%)	
Sex				
Female	4 (19.0)	3 (30.0)	7 (22.6)	0.672
Male	17 (81.0)	7 (70.0)	24 (77.4)	0.523
Current Smoker	6 (28.0)	4 (40.0)	10 (32.3)	0.692
Prior Smoker	13 (61.9)	5 (50.0)	18 (58.1)	0.531
Hypertension	20 (95.2)	10 (100.0)	30 (96.8)	0.552
Hyperlipidemia	15 (71.4)	7 (70.0)	22 (71.0)	0.561
Diabetes	2 (9.5)	0 (0.0)	2 (6.5)	0.563
CKD	2 (9.5)	0 (0.0)	2 (6.5)	0.574
CAD	10 (47.6)	4 (40.0)	14 (45.2)	0.584
COPD	8 (38.1)	5 (50.0)	13 (41.9)	0.601

ASA = American Society of Anesthesiologists; CAD = coronary artery disease; CKD = chronic kidney disease; COPD = chronic obstructive pulmonary disease; SD = standard deviation.

**Table 2:** Perioperative characteristics.

Variables	Group 0 No Thoracic Aortic Aneurysm Reintervention	Group 1 Thoracic Aortic Aneurysm Reintervention	All	*p*-Value
	n = 21 (67.7%)	n = 10 (32.3%)	n = 31 (100.0%)	
	n (%)	n (%)	n (%)	
Location of the aneurysm				
2 3	5 (23.8)	3 (30.0)	8 (25.8)	**0.041**
3 4	16 (76.2)	3 (30.0)	19 (61.3)	<0.212
2 3 4	0 (0.0)	4 (40.0)	4 (12.9)	**<0.001**
Spinal drain	5 (23.8)	5 (50.0)	10 (32.3)	0.390
Femoral arterial access	19 (90.5)	10 (100.0)	29 (93.5)	0.771
Iliac arterial access	1 (4.8)	0 (0.0)	1 (3.2)	1.000
Type endoleak				
Type I endoleak	0 (0.0)	5 (50.0)	5 (16.1)	**<0.001**
Type II endoleak	0 (0.0)	2 (20.0)	2 (6.5)	0.321
Type III endoleak	0 (0.0)	4 (40.0)	4 (12.9)	**<0.001**
	Mean ± SD	Mean ± SD	Mean ± SD	
No. of aortic devices	2.1 ± 0.8	2.2 ±1.3	2.1 ±0.9	**0.002**
Proximal neck leght (mm)	40.1 ± 19.0	30.2 ±13.1	35.15 ± 17.2	**<0.001**
Distal neck leght (mm)	17.8 ± 6.8	15.8 ± 5.1	18.7 ± 6.5	**<0.001**
Length of aortic coverage	202.9 ± 46.7	232.8 ± 93.1	213.2 ± 66.4	**0.002**

2-3-4: Location of the aneurysm within the thoracic aorta according to the Ishimaru classification system based on anatomic landmarks [25].

**Table 3:** Values of the CTA parameters at each follow-up time point.

Variables	Group 0 No Thoracic Aortic Aneurysm Reintervention	Group 1 Thoracic Aortic Aneurysm Reintervention	All	*p*-Value
	n = 21 (67.7%)	n = 10 (32.3%)	n = 31 (100.0%)	
	**T0 (mean ± SD)**			
Maximal aortic diameter (D), mm	65.1 ± 10.9	66.9 ± 10.7	65.6 ± 10.7	0.358
Aortic aneurysmal volume (AAV) mL	315.8 ± 134.3	303.1 ± 128.0	311.9 ± 130.2	0.888
Aneurysmal thrombus volume (ATV) mL	110.3 ± 57.9	125.5 ± 80.7	115.0 ± 64.7	0.832
Aneurysmal lumen volume (ALV) mL	183.4 ± 71.9	227.9 ± 79.6	197.2 ± 75.9	0.137
	**T1 (mean ± SD)**			
Maximal aortic diameter (D), mm	62.5 ± 11.1	66.1 ± 10.1	63.5 ± 10.9	0.326
Aortic aneurysmal volume (AAV) mL	303.7 ± 138.0	365.6 ± 207.4	322.0 ± 160.5	0.559
Aneurysmal thrombus volume (ATV) mL	116.9 ± 76.5	145.7 ± 65.9	125.5 ± 73.5	0.212
Aneurysmal lumen volume (ALV) mL	190.7 ± 72.1	277.7 ± 182.9	216.5 ± 119.4	0.202
	**T2 (mean ± SD)**			
Maximal aortic diameter (D), mm	57.6 ± 10.4	67.5 ± 10.5	61.0 ± 11.2	0.093
Aortic aneurysmal volume (AAV) mL	286.1 ± 117.4	436.4 ± 192.0	336.2 ± 159	0.080
Aneurysmal thrombus volume (ATV) mL	114.7 ± 64.7	187.6 ± 86.3	139.0 ± 78.7	**0.057**
Aneurysmal lumen volume (ALV) mL	191.5 ± 85.9	292.2 ± 116.3	225.1 ± 105.9	**0.048**
	**T3 (mean ± SD)**			
Maximal aortic diameter (D), mm	55.3 ± 12.6	67.3 ± 9.5	60.4 ± 12.7	**0.044**
Aortic aneurysmal volume (AAV) mL	263.4 ± 124.0	404.1 ± 183.0	321.4 ± 162.2	0.130
Aneurysmal thrombus volume (ATV) mL	82.1 ± 39.9	195 ± 86.7	128.8 ± 83.3	**0.013**
Aneurysmal lumen volume (ALV) mL	183.8 ± 90.8	251.0 ± 112.5	211.5 ± 102.7	0.223

SD = standard deviation; T0 = preoperative CTA; T1 = preoperative CTA to 1-year postoperative CTA; T2 = 2-year CTA; T3 = 3-year CTA.

**Table 4:** Diameter and volume annual growth rates at each time point (%/year).

		Group 0 No Thoracic Aortic Aneurysm Reintervention	Group 1 Thoracic Aortic Aneurysm Reintervention	All	*p*-Value
		n = 21 (67.7%)	n = 10 (32.3%)	n = 31 (100.0%)	
**T1 vs. T0**					
**D**	Mean (SD)	−4.19 (10.61)	−3.77 (10.15)	−4.08 (10.29)	0.932
	Median [Q1, Q3]	−2.99 [−6.54, 3.07]	0.81 [−11.83, 3.15]	−2.71 [−8.91, 3.08]	
**AAV**	Mean (SD)	−6.31 (6.83)	6.63 (4.49)	−2.83 (8.53)	**<0.001**
	Median [Q1, Q3]	−7.35 [−12.28, −0.87]	7.81 [3.04, 9.9]	−1.44 [−8.61, 2.01]	
**ATV**	Mean (SD)	32.78 (99.86)	42.77 (101.18)	35.47 (98.27)	0.831
	Median [Q1, Q3]	5.03 [−3.96, 29.88]	15.77 [−29.79, 61.81]	9.04 [−13.39, 37.8]	
**ALV**	Mean (SD)	1.09 (5.59)	1.96 (1.62)	1.32 (4.83)	**0.013**
	Median [Q1, Q3]	0.00 [−0.66, 0.75]	1.51 [0.90, 2.58]	0.31 [0.00, 1.23]	
**T2 vs. T1**					
**D**	Mean (SD)	−3.17 (6.69)	6.04 (9.72)	−0.61 (8.48)	**0.013**
	Median [Q1, Q3]	−1.33 [−6.24, −0.16]	2.72 [2.5, 7.78]	−0.64 [−3.9, 2.09]	
**AAV**	Mean (SD)	6.73 (29.7)	11.39 (14.63)	8.03 (26.03)	0.660
	Median [Q1, Q3]	−1.01 [−5.66, 3.45]	12.68 [3.56, 17.03]	0.33 [−4.92, 11.71]	
**ATV**	Mean (SD)	3.42 (32.55)	63.96 (87.93)	20.24 (57.84)	0.200
	Median [Q1, Q3]	1.01 [−16.77, 20.44]	28.87 [0, 98.08]	2.46 [−8.23, 27.5]	
**ALV**	Mean (SD)	10.52 (23.95)	6.58 (27.18)	5.77 (25.31)	0.401
	Median [Q1, Q3]	2.25 [−0.96, 24.50]	4.87 [−33.81, 11.50]	3.56 [−1.96, 21.87]	
**T3 vs. T1**					
**D**	Mean (SD)	−1.12 (1.9)	−0.2 (4.93)	−0.79 (3.15)	0.711
	Median [Q1, Q3]	−1.28 [−2.67, 0.11]	−0.06 [−3.29, 3.81]	−0.8 [−2.7, 0.64]	
**AAV**	Mean (SD)	−7.16 (18.2)	13.39 (16.91)	1.06 (20)	**0.046**
	Median [Q1, Q3]	−2.6 [−10.48, −0.92]	9.34 [−0.56, 22.35]	−0.92 [−2.7, 9.25]	
**ATV**	Mean (SD)	−12.97 (14.42)	34.37 (40.92)	5.96 (35.96)	**0.041**
	Median [Q1, Q3]	−11.48 [−22.76, −2.42]	31.29 [−1.87, 69.03]	−2.5 [−13.04, 3.83]	
**ALV**	Mean (SD)	−3.91 (28.28)	6.53 (30.26)	−0.43 (28.31)	0.751
	Median [Q1, Q3]	−0.98 [−5.53, 9.23]	0.34 [−14.26, 14.96]	−0.36 [−14.26, 9.63]	
**T3 vs. T2**					
**D**	Mean (SD)	−2.19 (3.76)	−0.2 (9.8)	−1.48 (6.26)	0.681
	Median [Q1, Q3]	−2.53 [−5.26, 0.23]	−0.13 [−6.46, 7.76]	−1.6 [−5.33, 1.29]	
**AAV**	Mean (SD)	−10.87 (28.74)	30.95 (39.78)	5.86 (38.56)	**0.056**
	Median [Q1, Q3]	−5.12 [−19.86, −1.84]	20.44 [−1.11, 49.74]	−1.84 [−5.33, 19.36]	
**ATV**	Mean (SD)	−7.76 (49.54)	27.15 (50.07)	3.88 (50.84)	**<0.001**
	Median [Q1, Q3]	−16.14 [−32.81, −1.84]	7.58 [−10.37, 47.93]	−10.37 [−20.82, 9.86]	
**ALV**	Mean (SD)	−3.91 (28.28)	6.53 (30.26)	−0.43 (28.31)	0.752
	Median [Q1, Q3]	−0.98 [−5.53, 9.23]	0.34 [−14.26, 14.96]	−0.36 [−14.26, 9.63]	

D = maximal aortic diameter; AAV = aortic aneurysmal volume; ATV = aneurysmal thrombus volume; ALV = aneurysmal lumen volume.

## Data Availability

The data presented in this study are available on request from the corresponding author. The data are not publicly available due to privacy restrictions.
